# Supplementary material for: Digital PCR-Based Characterization of a g10evo-epsps Gene-Specific Matrix Reference Material for Its Food and Feed Detection
Source: Foods. 2022 Jun 25;11(13):1888. doi: 10.3390/foods11131888 (PMC9266130; doi:10.3390/foods11131888)
Supplement: Supplementary file 1 [file foods-11-01888-s001.zip › foods-1760389-supplementary.pdf]

foods

Digital PCR-based Characterization of a g10evo-epsps Gene-Specific Matrix Reference Material for its Food and Feed Detection

Xiaoyun Chen [1], Huiru Yu [2], Pengfei Wang [3], Cheng Peng [1], Xiaofu Wang [1], Xiaoli Xu [1], Junfeng Xu [1,*] Jingang Liang [4,*] and Liang Li [5,*]

[1] State Key Laboratory for Managing Biotic and Chemical Threats to the Quality and Safety of Agro-products, Zhejiang Academy of Agricultural Sciences, Hangzhou 310021, China

[2] Zhejiang Normal University, Jinhua, 321001, China;

[3] Zhejiang University, Hangzhou, 310058, China;

[4] Development Center of Science and Technology, Ministry of Agriculture and Rural Affairs, Beijing, 100176, China;

[5] Institute of Quality Standard and Testing Technology for Agro-Products, Chinese Academy of Agricultural Sciences, 100081, Beijing, China;

* Corresponding author.

E-mail address: liliang@caas.cn (L. Li), liangjingang@agri.gov.cn (J.G. Liang).

Table S1

Determination of seed moisture content of ZUTS-33 soybean.

Sample	Moisture content（%）					Mean	SD	RSD
	1	2	3	4	5	（%）		（%）
ZUTS-33 soybean	7.05	7.47	7.36	6.94	7.56	7.28	0.003	3.69

Table S2

DNA quality extracted with different isolation kit.

Kit	OD_{260}/OD_{280}	OD_{260}/OD_{230}	DNA concentration /ng·μL^{-1}	DNA content /μg
CTAB	1.87±0.01	2.39±0.01	56.9±1.0	5.69±0.10
SDS	1.96±0.01	1.15±0.01	100.9±3.0	10.09±0.30
Rapid isolation	1.08±0.06	1.18±0.01	152.2±1.2	15.22±0.12

Table S3

Homozygote test result with ddPCR.

No of the Sample	Sample	Copies/20μLWell	Ratio	Result
1	*lectin*	4540	0.96	homozygote
	g10evo-epsps	4340		
2	*lectin*	4260	0.98	homozygote
	g10evo-epsps	4180		
3	*lectin*	3800	1.01	homozygote
	g10evo-epsps	3840		
4	*lectin*	4200	1.01	homozygote
	g10evo-epsps	4260		
5	*lectin*	4220	1.02	homozygote
	g10evo-epsps	4320		
6	*lectin*	4040	1.00	homozygote
	g10evo-epsps	4060		
7	*lectin*	4340	0.93	homozygote
	g10evo-epsps	4040		
8	*lectin*	3740	0.97	homozygote
	g10evo-epsps	3618		
9	*lectin*	3580	0.96	homozygote
	g10evo-epsps	3440		
10	*lectin*	3220	0.96	homozygote
	g10evo-epsps	3080		
11	*lectin*	4080	0.94	homozygote
	g10evo-epsps	3820		
12	*lectin*	4260	1.05	homozygote
	g10evo-epsps	4460		
13	*lectin*	3900	0.90	homozygote
	g10evo-epsps	3500		
14	*lectin*	4100	1.00	homozygote
	g10evo-epsps	4100		
15	*lectin*	4340	1.00	homozygote
	g10evo-epsps	4340		
16	*lectin*	4600	0.91	homozygote
	g10evo-epsps	4180		
17	*lectin*	3840	1.01	homozygote
	g10evo-epsps	3880		
18	*lectin*	3420	1.09	homozygote
	g10evo-epsps	3740		
19	*lectin*	3500	0.91	homozygote
	g10evo-epsps	3200		

20	*lectin*	3600	0.88	homozygote
	g10evo-epsps	3180		

Table S4

Results of *g10evo-epsps* homogeneity test (exogenous/endogenous copy number ratio)

Vails	*g10evo-epsps*					
	Experiments			Mean	SD	RSD
	1	2	3			
1	1.005	1.019	1.008	1.011	0.007	0.007
2	0.946	0.981	0.969	0.965	0.018	0.019
3	0.946	0.974	0.985	0.968	0.020	0.020
4	0.994	1.016	0.988	0.999	0.015	0.015
5	0.978	0.964	0.974	0.972	0.007	0.007
6	1.026	0.997	1.002	1.008	0.015	0.015
7	1.018	0.966	1.000	0.995	0.026	0.026
8	0.954	0.955	0.998	0.969	0.025	0.026
9	0.955	0.954	1.021	0.977	0.038	0.039
10	0.994	0.992	0.963	0.983	0.017	0.018
11	0.978	1.000	0.948	0.975	0.026	0.027
12	0.978	0.960	0.974	0.971	0.010	0.010
		Total Mean			0.983	
		Total SD			0.016	
		Total RSD（%）			0.017	

Table S5

Results of certified value in eight laboratories.

Lab	Test 1	Test 2	Test 3	Test 4	Test 5	Test 6	Test 7	Test 8	Mean	SD	RSD
1	0.98	0.97	0.96	0.97	0.98	0.96	0.97	0.96	0.970	0.01	0.99%
2	1.00	1.01	0.99	1.01	1.02	0.98	1.01	0.97	1.000	0.02	1.72%
3	0.99	1.01	1.02	1.01	1.02	1.00	1.02	1.00	1.009	0.01	1.07%
4	0.95	0.95	0.97	0.99	0.97	0.97	0.96	0.99	0.968	0.02	1.73%
5	0.96	1.00	0.97	0.99	0.93	0.98	0.98	0.94	0.968	0.02	2.47%
6	0.96	1.02	0.99	1.01	0.96	0.96	0.93	1.00	0.977	0.03	3.11%
7	0.95	0.98	0.92	0.98	0.96	0.96	0.95	0.99	0.960	0.02	2.26%
8	0.96	0.99	0.95	0.97	0.97	0.98	1.00	0.98	0.974	0.01	1.41%
Mean						0.978					
SD						0.018					
RSD						1.845%					

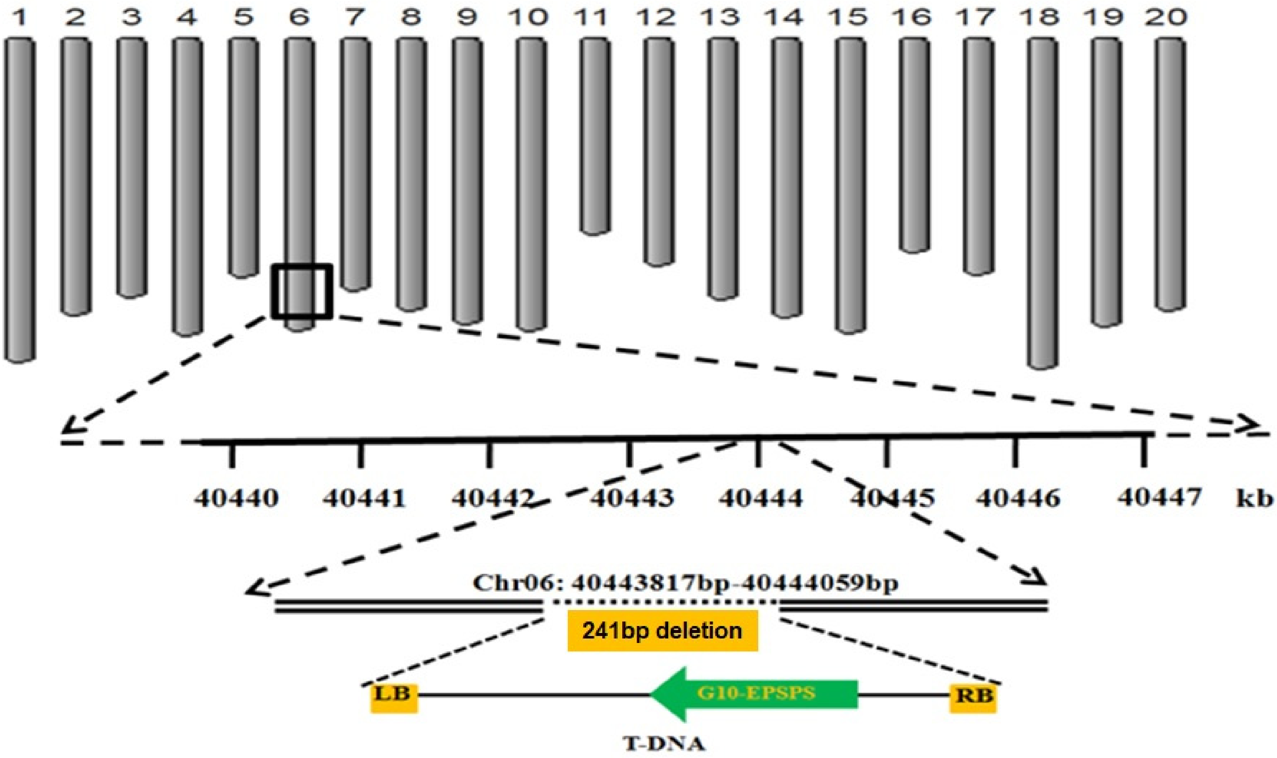

Figure S1. T-DNA structure and insertion site of the ZUTS-33 soybean

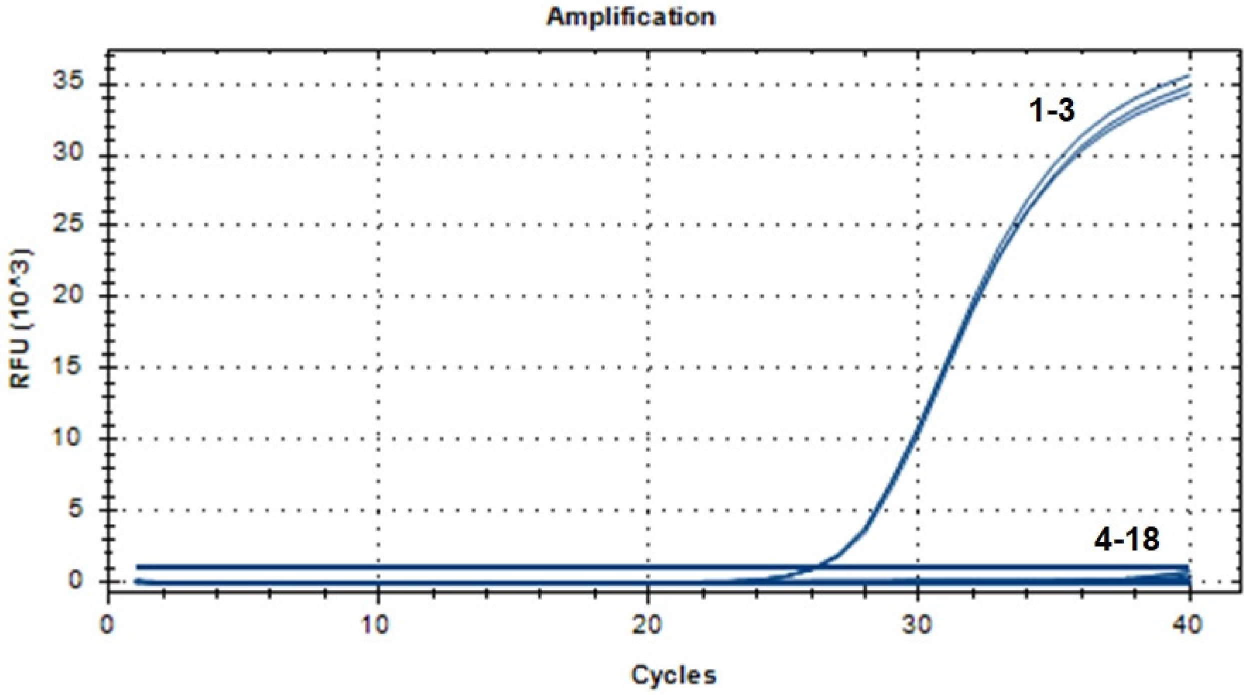

Figure S2. ZUTS-33 soybean event specific real time PCR amplification curve.

Line 1-3, three duplicates of GM soybean containing 0.5 ng; Line 4-6, three duplicates of mixed GM maize (Bt11, Bt176, MON810, MON863, GA21, NK603, T25, TC1507, MON89034, MON88017, 59122, MIR604, 3272, MON87460, MIR162, DAS40278-9, Shuangkang 12-5, IE09S034, C0030.3.5, C0010.3.7, 4114, MON87427, 5307, the content of each sample is 1%); Line 7-9, three duplicates of mixed GM soybean (GTS40-3-2, MON89788, CV127, A5547-127, A2704-12, 305423, 356043, MON88302, 73496, MON87769, MON87705, FG72, DAS68416-4, the content of each sample is 1%); Line 10-12, three duplicates of mixed GM rice (TT51-1, Kefeng 6, Kemingdao, M12, Kefeng 8, Kefeng 2, G6H1, T1C-19, the content of each sample is 1%); Line 13-15, three duplicates of mixed GM cotton (MON531, MON1445, MON15985, LLCOTTON25, MON88913, GHB614, COT102, the content of each sample is 1%); Line 16-18, three duplicates of mixed GM canola (Ms1, Ms8, RF1, RF2, RF3, T45, oxy235, Topas19/2, MON88302, 73496, the content of each sample is 1%).
